# Digital and Mobile Technologies to Promote Physical Health Behavior Change and Provide Psychological Support for Patients Undergoing Elective Surgery: Meta-Ethnography and Systematic Review

**DOI:** 10.2196/19237

**Published:** 2020-12-01

**Authors:** Anna Robinson, Umay Oksuz, Robert Slight, Sarah Slight, Andrew Husband

**Affiliations:** 1 School of Pharmacy Population Health Sciences Institute Newcastle University Newcastle upon Tyne United Kingdom; 2 Population Health Sciences Institute Newcastle University Newcastle upon Tyne United Kingdom; 3 Newcastle upon Tyne Hospitals NHS Foundation Trust Newcastle upon Tyne United Kingdom

**Keywords:** mobile health, mHealth, healthy lifestyle, bariatric surgery, cancer, orthopedic procedures, qualitative research, systematic review, telemedicine, mobile phone

## Abstract

**Background:**

Digital technology has influenced many aspects of modern living, including health care. In the context of elective surgeries, there is a strong association between preoperative physical and psychological preparedness, and improved postoperative outcomes. Health behavior changes made in the pre- and postoperative periods can be fundamental in determining the outcomes and success of elective surgeries. Understanding the potential unmet needs of patients undergoing elective surgery is central to motivating health behavior change. Integrating digital and mobile health technologies within the elective surgical pathway could be a strategy to remotely deliver this support to patients.

**Objective:**

This meta-ethnographic systematic review explores digital interventions supporting patients undergoing elective surgery with health behavior changes, specifically physical activity, weight loss, dietary intake, and psychological support.

**Methods:**

A literature search was conducted in October 2019 across 6 electronic databases (International Prospective Register of Systematic Reviews [PROSPERO]: CRD42020157813). Qualitative studies were included if they evaluated the use of digital technologies supporting behavior change in adult patients undergoing elective surgery during the pre- or postoperative period. Study quality was assessed using the Critical Appraisal Skills Programme tool. A meta-ethnographic approach was used to synthesize existing qualitative data, using the *7 phases of meta-ethnography* by Noblit and Hare. Using this approach, along with reciprocal translation, enabled the development of 4 themes from the data.

**Results:**

A total of 18 studies were included covering bariatric (n=2, 11%), cancer (n=13, 72%), and orthopedic (n=3, 17%) surgeries. The 4 overarching themes appear to be key in understanding and determining the effectiveness of digital and mobile interventions to support surgical patients. To successfully motivate health behavior change, technologies should provide motivation and support, enable patient engagement, facilitate peer networking, and meet individualized patient needs. Self-regulatory features such as goal setting heightened patient motivation. The personalization of difficulty levels in virtual reality–based rehabilitation was positively received. Internet-based cognitive behavioral therapy reduced depression and distress in patients undergoing cancer surgery. Peer networking provided emotional support beyond that of patient-provider relationships, improving quality of life and care satisfaction. Patients expressed the desire for digital interventions to be individually tailored according to their physical and psychological needs, before and after surgery.

**Conclusions:**

These findings have the potential to influence the future design of patient-centered digital and mobile health technologies and demonstrate a multipurpose role for digital technologies in the elective surgical pathway by motivating health behavior change and offering psychological support. Through the synthesis of patient suggestions, we highlight areas for digital technology optimization and emphasize the importance of content tailored to suit individual patients and surgical procedures. There is a significant rationale for involving patients in the cocreation of digital health technologies to enhance engagement, better support behavior change, and improve surgical outcomes.

## Introduction

### Background

The introduction of digital technologies has influenced many aspects of modern living, including health care [1,2]. Digital health technologies (eg, wearable activity trackers and mobile phone apps) provide opportunities for effective patient care. They can improve communication between health care providers [3], facilitate connectivity with clinicians and peers [4,5], enable remote health monitoring [6], and empower patients to play an active role in their long-term care [3,7-9].

In the context of elective surgeries, there is a strong association between preoperative physical and psychological preparedness and improved postoperative outcomes [10-12]. More specifically, improvements in physical activity levels [13], dietary intake [14], and smoking cessation [15] have been linked to improved recovery after surgery, reduced risk of complications, better tolerance of postsurgical adjunctive treatment, and prevention of long-term disease [16-19].

Although health behavior changes made in the pre- and postoperative periods can be fundamental in determining the outcomes and success of elective surgeries [19-21], there are variable amounts of support and education currently provided to patients undergoing elective surgery to motivate these health behavior changes [22-24]. A recent study evaluating patient attitudes to health behavior changes found that although preoperative patients understood the health benefits of improved behaviors, they lacked the confidence to make such changes without intervention or support [19]. Many physical and mental health interventions offered in elective care pathways use face-to-face, in-person delivery for individuals or small groups of patients. Such approaches are resource- and time-intensive for staff already working in high-pressure health care sectors [25-27]. In addition, geographic isolation, travel costs, and the time burden of attending classes can all negatively affect patient engagement with postoperative appointments [28,29]. Understanding the potential unmet needs of patients undergoing elective surgery is central to motivating health behavior changes. Integrating digital technologies within the elective surgical pathway could be one strategy to remotely deliver behavioral change advice and lifestyle support, consequently improving patient engagement and postoperative success rates [12,30].

### Approach

This review uses a meta-ethnographic approach to analyze and synthesize qualitative findings. Meta-ethnography was originally developed by Noblit and Hare [31], but it has recently been used in health care–based social science research by Britten et al [32], Campbell et al [33,34], Pound et al [35], and others. It is an inductive and interpretive approach involving the translation of papers into one another. Meta-ethnographies encourage researchers to understand and transfer ideas, themes, and metaphors across different studies to gain a deeper understanding or to inform the development of broader concepts [31,36].

There are still unanswered questions relating to the optimization of digital technologies to support patients undergoing elective surgery, especially in the cohorts of bariatric, cancer, and orthopedic surgery. We seek to synthesize findings from existing qualitative research to determine whether digital technologies are effective in supporting patients undergoing elective surgery to change their health behaviors, specifically focusing on physical activity, weight, dietary intake, and mental health support (eg, cognitive behavioral therapy).

## Methods

This meta-ethnographic systematic review is registered with PROSPERO (registration number CRD42020157813) and has been conducted in accordance with the PRISMA (Preferred Reporting Items for Systematic Reviews and Meta-Analyses) guidelines (Multimedia Appendix 1).

### Search Strategy and Information Sources

 A comprehensive and systematic literature search was conducted in October 2019 across 6 electronic databases: MEDLINE, EMBASE, CINAHL, PsycINFO, Web of Science, and Scopus. No limit on the publication date was applied. Additional papers were identified via gray literature using Google Scholar, and we manually searched the bibliographies of all included studies. A full list of search terms is included in Multimedia Appendix 1.

### Eligibility Criteria

This meta-ethnography focused on elective surgical procedures, specifically bariatric, cancer, and orthopedic surgeries. Patients undergoing these elective procedures may have improved surgical outcomes owing to pre- and postoperative health behavior changes and therefore can benefit from the support of digital health technologies. Acute, unplanned surgeries and emergency trauma procedures were excluded from this review.

Only the studies that had encompassed the use of digital health interventions to support behavior changes (such as weight changes, dietary intake, physical activity levels, and/or mental health strategies) in adult patients undergoing elective surgery (>18 years) during the pre- or postoperative period were included. There were no restrictions placed on participants’ sex, ethnicity, or nationality. The included studies must be qualitative or mixed method studies containing a significant qualitative component to analyze participant perspectives (eg, patient interviews or focus groups).

Exclusion criteria included studies employing behavior changes achieved by nondigital interventions; participants who were not scheduled to undergo an elective bariatric, cancer, and orthopedic surgery; studies where the intervention was mainly focused on perspectives of health care professionals; nonqualitative studies (eg, quantitative studies, systematic reviews, or protocols); and studies in languages other than English.

### Selection of Eligible Studies

**Figure 1:**
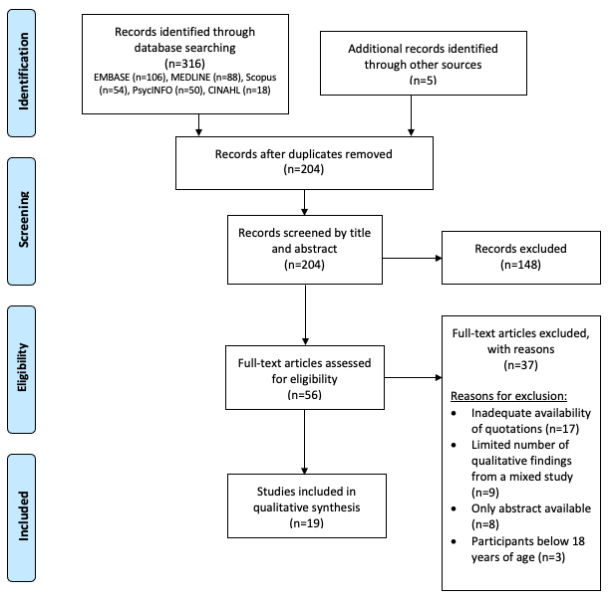
PRISMA (Preferred Reporting Items for Systematic Reviews and Meta-Analyses) flow diagram of included studies.

Two authors (UO and AR) reviewed the titles and abstracts from the database search. Full texts were retrieved for articles that met the inclusion criteria and for those that could not be rejected with certainty. Two authors (UO and AR, with an agreement rate of 94.7%) independently screened the full texts of eligible articles. Disagreements (on 3 of the 56 articles) were resolved through discussion with a third reviewer (AH). Figure 1 shows a PRISMA flowchart for the study selection process.

### Reading, Data Extraction, and Quality Appraisal

Two authors (UO and AR) closely read and re-read the included studies to ensure close familiarity with the work. Data extraction was performed across the full primary study (by UO and AR) [36] and carried out using a customized data extraction form, including the study and author details, method of intervention delivery, population data, inclusion criteria, and original quotes and/or concepts developed by the authors of primary studies (within their original context). Both authors worked independently before comparing their work; disagreements were resolved through discussion with a third reviewer (AH) where necessary. Quality appraisal was conducted independently by UO and AR using the Critical Appraisal Skills Programme (CASP) questions to understand qualitative research [37]. No papers were excluded on the grounds of quality.

### Analysis and Interpretive Synthesis

Meta-ethnographic approaches were applied to this review, as determined by the 7 phases of meta-ethnography by Noblit and Hare [31]: (1) *getting started*, (2) *deciding what is relevant to the initial interest*, (3) *reading the studies*, (4) *determining how studies are related*, (5) *translating the studies into one another*, (6) *synthesizing translations*, and (7) *expressing the synthesis*.

The findings (concepts and metaphors) from the primary studies were compared to determine how they are related. Noblit and Hare [31] suggested that phase 5, where findings are translated into one another, follows something like “one case is like another, except that...”. This phase of a meta-ethnographic approach is termed “reciprocal translation,” and it enables the development of themes and subthemes for interpretive synthesis [31,33]. According to this, we developed 4 overarching themes (or third-order constructs) and subsequent subthemes that were consistent with the original results but also extended beyond them.

When translating the studies into one another to develop themes (and subthemes), we arranged each paper chronologically and compared the themes from paper 1 with those of paper 2, then those of paper 2 with those of paper 3, and so on. As we compared each study, we grouped similar themes and continually reviewed and refined them until they were coherent and distinctive. Two reviewers (UO and AR) were involved in the study translation at all times; however, if agreement was not reached between these, discussions with a third author (AH) helped to establish a consensus.

To adhere to recommendations for conducting meta-ethnographies, we used the term “theme” to describe the third-order construct and subthemes to describe third-order construct subthemes [36]. The development of these overarching themes enables meta-ethnographies to delve further into a topic as compared with a traditional systematic review and contribute new insights to the literature [32].

We report on the overall effectiveness of digital health technologies to support behavioral change in patients undergoing surgery through 4 established themes: (1) motivational support, (2) patient engagement with interventions, (3) the facilitation of peer networking, and (4) intervention specificity to meet patients’ individual needs.

## Results

### Search Results

A total of 316 citations were retrieved from the database searches. A total of 5 additional records were identified through gray literature and searching references manually from relevant studies. Following the removal of duplicates (n=112), 204 papers were screened, of which 148 were excluded based on their titles and abstracts. A total of 56 full-text papers were assessed for eligibility; 38 of these were excluded due to reasons detailed in the PRISMA flowchart in Figure 1. The remaining 18 studies were included in this meta-ethnographic systematic review; of these, 68% (n=13) were qualitative and 32% (n=7) were mixed methods studies.

### Study Characteristics

All 18 included papers were published between 2013 and 2019. The study was conducted in 8 different countries: United States (n=6) [38-43], United Kingdom (n=3) [44-46], Canada (n=3) [47-49], Australia (n=2) [50,51], Ireland (n=1) [52], Norway (n=1) [53], South Korea (n=1) [54], and China (n=1) [55].

The 18 studies covered 3 different surgery types: bariatric (n=2, 11%), cancer (n=13, 72%), and orthopedic (n=3, 17%) surgeries. Further study characteristics, including the method of intervention delivery and original themes extracted from the study, are detailed in Multimedia Appendix 1 [35,37-55].

A total of 3 main intervention delivery methods were identified in the 18 included studies. These included internet-based interventions (eg, emails, e-platforms, virtual reality, tele-rehabilitation) [38,40,42,44,46,48,49,53,54], mobile phone–based interventions (eg, text messages, smartphone apps) [39,45,55], and wearable interventions (eg, activity trackers) [43,47,50-52]. Only 1 study reported the use of a combination of 2 intervention methods (dual approach), including wearable- and phone-based interventions [41].

### Study Quality

Multimedia Appendix 1 contains details of the quality appraisal conducted using the CASP tool for qualitative studies. Of the included studies, Shaffer et al [38], Phillips et al [39], Alberts et al [40], and Argent et al [52] were identified as having the highest quality.

### Findings: Reporting Outcomes, Synthesizing Translations, and Developing Themes and Subthemes

**Figure 2:**
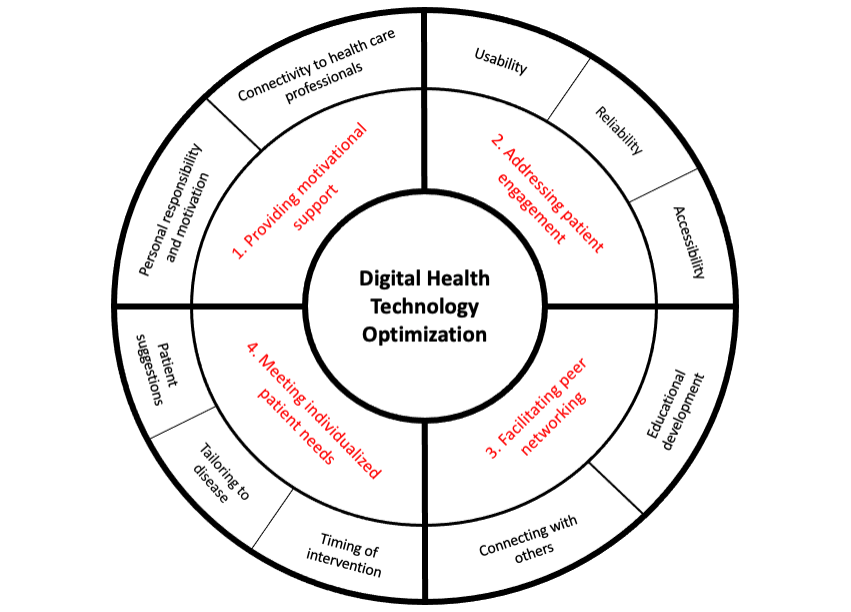
Developed themes and subthemes for Digital Health Technology Optimization. The inner band on the diagram (red text) represents the 4 overarching themes developed by this review, and the outer band details the subsequent subthemes (black text).

Multimedia Appendix 1 presents the metaphors and patients’ perspectives from each of the included studies. Reciprocal translation and refutation of these concepts enabled the development of 4 overarching themes and subthemes for this meta-ethnography; they are outlined in Figure 2. The 4 overarching themes and subthemes appear to be key in understanding and determining the effectiveness of digital and mobile health interventions to support behavior change in patients undergoing surgery.

The qualitative data synthesis can be found in Multimedia Appendix 1, with each table representing one of the 4 overarching themes. These tables showcase examples of direct quotations (first-order constructs) from study participants, the authors’ interpretations of the original findings from the included studies (second-order constructs), and our interpretation (third-order constructs) and subthemes.

#### Providing Motivational Support

##### Personal Responsibility and Motivation

Certain features of digital and mobile health technologies increased patients’ self-awareness and motivation for physical activity. Patients reported that wearable activity trackers (termed wearables) made them more aware of the importance of physical activity and helped them monitor their sedentary behavior levels, which acted as a source of motivation to engage with positive behavior change [43,47,51]. Self-regulatory features of the wearables, including goal setting and performance feedback, facilitated personal fulfilment and gave orthopedic and cancer surgical patients a sense of control and accomplishment [50-52,54]:

Seeing your progress, I think is very important. Seeing measurable progress, whether it’s in calories burned, or minutes, or meeting a percentage of your goal [39].

set goals, like mid-week if I wanna hit 150 [minutes] I should be at half that [...] and the application is on my phone and I can see what I’ve done [...] so it’s really easy to track how well you’re doing or how well you’re not doing [47].

Patients perceived feedback and text messages as methods of encouragement, motivation, and support [39]. Gell et al [41] noted that health coaching, when offered alongside daily wearable use, provided patients undergoing cancer with an increased sense of self-importance, encouraging the maintenance of physical activity:

If you get to say 8000 [steps] in a day, you’re motivated to do those extra 2000 because you’re so close. It’s like “Why would I stop now?” I might as well keep going [50].

However, findings reported the potential for this to shift to *fear of failure* with nonadherence, where prompts or reminders could turn into negative judgments [47]:

for now, I don’t wanna [sic] be judged or evaluated or anything else… and then that will change…It’s just a case of you get tired of

judgment

##### Connectivity to Health Care Professionals

Patients undergoing bariatric surgery reported an increased feeling of accountability and responsibility to adhere to treatment plans when they were being monitored through digital or mobile health technologies [53,56]. Cancer and orthopedic surgical patients reported benefits of enhanced connectivity with members of their clinical team, including the provision of timely and personalized feedback [46,55] and the availability of instant communication for information-seeking needs [48,55]. A lower threshold for information seeking via digital technologies was reported by patients undergoing bariatric surgery, with sensitive questions being asked more readily [53]. Das and Faxvaag [53] evaluated the impact of an online forum on interactions between health care professionals and patients undergoing bariatric surgery. The authors recognized that the connectivity provided an easier access to evidence-based advice as well as offered a convenient and geographically independent platform to promote patient engagement [55]:

I could ask questions through the app regarding my medical condition. I could upload the lab results through your program. Then I received corresponding advice from experts. I felt followed up. When I knew more about my medical condition, I felt more likely to gain control of my life.

I live far away from the hospital and I have no doctor close to me. When I had questions about my medical condition, I could not find the answer in the internet. Then I asked questions through the app. Aha, the professor or expert responded.

Although this increased connectivity with health care professionals was reported to be beneficial in supporting postoperative recovery, patients undergoing cancer still felt that technologies should not replace traditional face-to-face appointments with clinicians. Concerns were raised by this cohort, with patients reporting that they may miss out on vital interactions, such as displays of empathy, which come from face-to-face communication.

#### Addressing Patient Engagement

##### Usability

Simplicity and ease of use were identified as prerequisites for effective engagement with digital and mobile health technologies in cancer and bariatric studies [39,40,42,43,47,48,52]. Patients in these studies reported the importance of feeling relaxed and at ease while using technology [40]. It is important to avoid complex or difficult interventions that may decrease a user’s enjoyment [54]:

Well it was very simple. It was straightforward. It wasn’t complicated…like going through chemo you have kind of a brain scramble and… just the simplest things you can’t wrap your brain around sometimes [40].

I would say the most important thing is the ease of use, the simplicity of it, because if it’s cumbersome I will not use it [39].

Some participants undergoing varied cancer surgeries encountered technical difficulties while operating and synchronizing devices, which affected their rates of engagement [43,50,55]:

Largely, I'm not wearing it because it doesn‘t interact with my computer very easily...why bother? I just go use my manual step counter [43].

In studies evaluating wearable technologies, *wearability* was deemed important with references to comfort and style improving user engagement before and after cancer and orthopedic surgery [50-52]:

I didn’t like wearing it at night. I didn’t feel comfortable [50].

I had the Polar first [...] I thought it was quite heavy and quite clunky but then I had the two Garmins and in the end I decided that was my favourite even though it was heavier [50].

##### Reliability

The reliability of digital and mobile health technologies also affected engagement; patients recognized inaccuracies, which resulted in a lack of trust in the interventions and poor adherence to postoperative physical activity guidance [43,51,55]:

It seemed to register less activity than I felt I actually did because it was only measuring steps, and I was doing more than steps. I was lifting. I was bending. I was twisting. I was doing all that other sort of stuff [43].

The app sometimes was unstable. It didn’t work when I tried to open it. I contacted with someone in the hospital and reinstalled the app. Then I could log in. However, after a period of time, I couldn’t open the app again. Finally, I gave up using your program. I haven’t log in for the recent month 55.

##### Accessibility

The accessibility that digital and mobile health technologies offer was perceived as beneficial by all surgical cohorts, particularly if participants were geographically, economically, or functionally isolated [40,42,48]. Digital interventions reduced the time and cost of travel to clinics, an advantage over facility-based interventions [40,48]:

I really like it (telerehabilitation). I found it fantastic...you know, just the fact of not having to travel when we are in pain (...) I adored it [48].

Well, definitely the availability of it to anybody, no matter where you live. I know we work with a lot of rural people and after they’re done here, they don’t want to travel for more therapy or whatever, so something that they can do at home [40].

#### Facilitating Peer Networking

##### Educational Development

By building a peer network, digital and mobile health technologies provide patients undergoing enhanced access to knowledge and support and, as a result, can motivate health behavior changes to improve surgical outcomes. Informational support delivered by peers was perceived as useful and relatable by patients undergoing bariatric and cancer surgeries. Patient satisfaction and reassurance were reported from the sharing of personal anecdotes and advice after bariatric surgery [49]. Strategies addressing preoperative concerns and the challenges of adhering to surgery guidelines were also shared [42,49,53]:

[Product name]... this is odourless and tasteless and does not clump. You can add it to hot or cold... or just sprinkle over your food. One tablespoon equals a scoop of Whey and has 30 grams of protein. It is approved by [Medical Association] and has 96% absorption... [49]

I think it is more enjoyable to write a “diary” that everyone can read and comment on. I like to get feedback on how I do things, what I eat, and thoughts that I have about the surgery and about life after the operation, so here comes a little of everything...Hope you will read and comment [53].

...You may want to pick up a pill crusher and a pill splitter in the drug store. The large pills such as calcium citrate, I had to crush and mix with drink in order to take them... [49]

##### Connecting With Others

In addition to informational support, digital and mobile health technologies and online forums provided emotional support to patients. Studies referred to the benefits of patients undergoing cancer communicating with others who have had the same surgical procedures or experience with the same disease-related condition [40,42,50]. Peer interactions have helped patients overcome feelings of loneliness and improved individual mental well-being [40,45,55]. In preoperative peer forums, encouraging messages have motivated patients undergoing cancer surgery and bariatric surgery to lose weight and adhere to physical activity and dietary guidelines before surgery [40,42,45,49,50,55]:

You know that you’re not alone, but when your feelings are validated just by reading someone’s story, I mean that is everything [40].

It is so important to get in touch with people who went through the same thing as you have. [...] I think that if an app for cancer survivors had a forum on it as a part of the application to motivate each other, that would be amazing [45].

I feel better to talk to someone who is in similar situations. Cancer is not a good thing. If I always think about breast cancer alone at home, it is so easy for me to feel bad. I didn’t feel alone when I talked with peers through your program [55].

#### Meeting Patients’ Individual Needs

##### Timing of the Intervention

According to patients undergoing surgical cancer, initiating and tailoring the content of a digital or mobile intervention appears to be essential to determine its effectiveness to motivate behavior change. Two papers discussed the optimal time to start an intervention within a surgical journey; some patients undergoing cancer suggested initiation should be during the preoperative period to enable preparedness and understanding of processes [42], whereas others favored postoperative provision [38]:

I wish I would have had something like this when I was first diagnosed... I can see this tool being useful in answering questions that have not come to mind [38].

I had more trouble with sleep issues early on at diagnosis and in between surgeries, so it would have been helpful for me to have enrolled in the program earlier [42].

This cohort reported a preference to start with interventions once adjuvant chemotherapy was completed, citing treatment burden and side effects as factors for disengagement at this time. Immediate postoperative issues, such as fatigue, were also noted to impact early engagement rates [45]. However, some patients appreciated low-effort strategies during the surgical journey to manage symptoms and improve relaxation [38]:

The very end of your treatment when you finished your chemo and...the doctor says “Ok, see you in six months.” That would be the time to offer it. “Cause you feel so unwarned [sic].”

Interestingly, there was a general agreement among cancer patients that the best time to begin an intervention is “when you recognise that you have a problem ... and that you want help.”

##### Tailoring According to the Disease

Participants with surgical cancer also expressed a desire for intervention tailoring according to their changing physical and psychological health needs [38,39,42,45,47,50,55], focusing on information on their disease and surgical type [39,42,55]. Puszkiewicz et al [45] noted preferences for individualization of digital interventions according to patient lifestyles rather than a disease on the whole:

The issues I might have as a colorectal cancer survivor are very different from the ones than someone who had breast cancer or prostate cancer.

Anyone with any condition could use this program, which is beneficial, but it could be more beneficial [...] more tailored to the type of cancer or disease you had, to your lifestyle and fitness goals. I think it could be more fine-tuned to your circumstances, lifestyle, then that would be really helpful.

In the virtual reality–based rehabilitation study, participants expressed positive views on the personalized task difficulty, where the varied level of difficulties helped them to choose the exercise program according to their needs, and subsequently increased their satisfaction with the intervention [45].

##### Patient Recommendations

Participants across all 3 surgical cohorts suggested design and technical improvements for the future development of digital and mobile interventions. Although these varied depending on the delivery method, a user-centered design was identified as a key solution to enhance and maintain engagement and to motivate behavior changes [39]:

I think that it needs to be aimed towards survivors. That would be the first component. There’s a lot on the Internet that gives you a lot of exercises but it’s not aimed towards survivors.

Patient-reported design improvements for wearables included higher accuracy of the devices [39,50] different aesthetics (such as the tone of the prompt and color scheme) [47,50,51] and personal goal setting [50]:

So I'll give you a case. I filled my laundry, and it’s logged I walked 2,000 steps. I did not walk 2,000 steps 43.

I’d get a little vibration to say let’s go do 250 steps, it was much more polite than MOVE 51.

I like that the colour scheme was NOT pink! 48

In online forums for patients undergoing bariatric surgery or cancer surgery, fear of self-disclosure was a recognized barrier that affected user engagement. Full anonymity would make it easier to share sensitive issues and ask difficult questions [49,55]:

On other forums, even though you don’t have your name, with a nickname, you can find out who the person is anyway. You have to be very careful if you want to be anonymous 53.

Participants also suggested adding *search* tools to locate information and save time [55], as well as the inclusion of diet recommendations and/or self-monitored food intake [55]:

The program can be improved by adding search engine in the Learning forum. If I search for “nausea” then all the knowledge related to nausea will come out. Search engine will help save my time [55].

We are in a dilemma on what we should eat. The apps can provide detailed information on food choice, the time of food intake, the cooking methods, etc...Such practical information would be very helpful.

Older users appeared more likely to experience usability issues with interventions [55]. To overcome this, patients reported preferences for *open access* so that family members or caregivers can offer support [55]:

I was overwhelmed by the information each time I opened it.

Some people, like me, 40 or 50 years old. Well, this group believe the apps is a little bit troublesome. They feel challenged to use the new technology... If this program can be available for their family members, such as their son or daughter, it would be helpful.

Many women with breast cancer come from the countryside. They are illiterate, or they cannot read and speak Mandarin... if you can open the program to other family members who can read and convey the knowledge to the women, they would also benefit.

## Discussion

### Principal Findings

To our knowledge, this is the first meta-ethnographic systematic review examining the effectiveness of digital and mobile technologies to support health behavior change in patients undergoing elective surgery. Using reciprocal translation, our findings indicate 4 themes that appear to be key in determining intervention effectiveness to support health behavior change in patients undergoing surgery: (1) providing motivational support, (2) addressing patient engagement, (3) facilitating peer networking, and (4) meeting individualized patient needs. Future studies could use these findings to inform future design frameworks for specific surgical cohorts while embracing digital transformations in health care.

### Limitations

Although meta-ethnographies offer an opportunity to synthesize findings to develop new or deeper understandings on a subject, the process is largely interpretive [31]; other conclusions from the same included studies may be possible but still equally as valid. It is also important to note that the focus of this meta-ethnography was solely elective cancer, bariatric, and orthopedic surgeries, and as such, the meaning of our findings may not be generalizable for acute surgeries or other specialties.

### Comparisons With Previous Work

Digital and mobile technologies act as a catalyst to engage with healthy behaviors, such as loss of weight, improved dietary intake, and increased physical activity levels. Messages of positive reinforcement were viewed as useful, particularly when tailored to an individual’s surgical type and readiness to make behavioral change. Existing literature suggests that individualized goal setting helps combat sedentary behavior [57-59]; personalized feedback and messages of encouragement provide a sense of accomplishment [25,39]; and visual tracking of step count has been reported as motivational [50,58]. Recent contributions to the health behavior change literature have cited the importance of empowered patient-centered strategies and use of self-regulation [60] and self-determination theoretical frameworks [61,62] to improve patient motivation. Digital technologies underpinned by behavior change theory can promote a proactive and holistic strategy to influence behavior change in a modern health care system such as the UK National Health Service [21].

In the context of patients undergoing surgical cancer, internet-delivered cognitive behavioral therapy (iCBT) was associated with numerous benefits [40,44]. Following digital intervention usage, there have been improvements related to fatigue, sleep [63], depression [64], and psychological distress [65]. In addition, our findings suggest that iCBT can also educate participants around various coping strategies to manage fears of treatment and disease recurrence [40].

Technologies enabling connectivity to health care professionals have been positively acknowledged. Two-way telemedicine consultations, emails, and text message discussions facilitated improved information delivery, real-time goal setting, psychosocial outcomes, and decision making [66-68]. Participants felt motivated, reassured, and encouraged to adhere to postoperative advice through remote monitoring. Having access to health care professionals *behind a screen* also helped patients overcome their personal barriers and raise unmet needs beyond routine clinical questioning [53,67]. From the perspective of clinicians, digital and mobile health technologies provided them with a means to monitor patient progress, which enabled individualized advice to be given to reinforce beneficial behavior change [43,69].

Despite the benefits of digitally enabled communication, it is worth considering social norms with patient-professional relationships [70]. For some, the continuity of face-to-face appointments is essential to provide empathetic interaction and social support [70,71]. Empathy, rapport, and compassion through nonverbal behavior and body language is difficult to establish when communicating digitally. Despite this, Kairy et al [48] reported close relationships and trust between the therapist and patients when communicating *via* telerehabilitation. Perhaps, complementing traditional face-to-face appointments with digital health interventions could be a way to maintain patient-professional relationships.

Usability has been reported as a key determinant to induce and maintain health behavior change, where interventions should be easy to use as well as aesthetically and visually appealing. Patient preferences should be taken into account when it comes to the design and tailoring of interventions [50,72,73]. It is worth considering ways to overcome digital health literacy barriers to further promote usability and engagement. Additional technical support might be beneficial when targeting older adult populations to increase their engagement and thus better support health behavior change [50,74].

One reported advantage of digital interventions is the accessibility they offer [29,51,75]. Postoperative breast cancer survivors living in rural settings experienced greater depressive symptoms compared with those with shorter commutes owing to the long travel distances required to access health services [76,77]. Where telerehabilitation was implemented for postoperative orthopedic follow-ups, participants reported improved continuity of care with the same physician and improved ability to control the timing of appointments and intensity of the rehabilitation service [78].

In addition to bridging access to health services, digital and mobile health technologies are being increasingly used as networking and peer-support tools. Patients undergoing similar procedures or diagnosed with similar conditions are able to communicate and share personal experiences and coping strategies with others [53]. Peer support and behavior change have been previously reported in elective care [50,79-82], where increased social support and decreased patient isolation are associated with postsurgical success [81,83]. Although digital technologies offer opportunities to interact with peers on an educational level, concerns have been raised about the accuracy and credibility of shared information [79,84-86]. Health care professionals should caution patients when interpreting discussions on forums or online groups, given the potential detriments that may arise from following inaccurate information [53,86,87].

The optimal time point in the surgical pathway to initiate digital and mobile technologies remains uncertain, with findings suggesting that this may vary depending on the type of surgical group. Despite this, what remains clear is the potential benefit of capitalizing on a *teachable moment* to empower and educate patients about the underlying benefits of health behavior changes [88-90]. Evidence suggests that preoperative interventions based on education of lifestyle changes are significantly more effective in managing postoperative complications and patient expectations [91].

Our research has synthesized numerous design considerations that should be examined when producing future interventions to support patients undergoing surgery. It was found that internet-based interventions may benefit from adding a *search* tool to locate target information [38], the comfort of wearable technologies should be addressed [43,50], and negative connotations with using the color pink for patients undergoing cancer builds on the *cancer culture divide* [92]. The possible benefits of incorporating open-access features within interventions were also discussed. Previous research has shown that opening care access, to include relatives or caregivers, provided patients undergoing an increased sense of pre- and postoperative support [93-95]. This approach has strengthened bonds with family members, improved patient experience, resulted in effective engagement with digital interventions, and therefore supported superior outcomes in lifestyle changes [96-98]. This review synthesizes existing research to gain a deeper understanding of the ways in which digital tools can support elective surgical cohorts and identify key design features that support elective surgical patients to change their health behaviors, and thus have a greater impact on postoperative health. Considering the rapidly progressive nature of digital health interventions and digital assistive technology research, cocreation of a person-centered digital support network may help surgical patient cohorts to benefit from pre- and postoperative behavior changes on both a short- and long-term basis [19,99].

### Conclusions

This meta-ethnographic synthesis developed 4 key themes that are important in determining the success of technologies to support behavior change. Our novel findings have the potential to influence the future design of patient-centered digital and mobile health technologies. This study demonstrates the important role of digital tools in the elective surgical pathway; not only can they help to motivate physical behavior change, such as improved activity levels and dietary intake, but they can also successfully provide psychological support. By synthesizing patient-informed suggestions, we have identified key areas for improvement, both to meet the general desires of patients undergoing surgery and to meet more specialized surgery-specific needs throughout the perioperative pathway. In particular, digital technologies should optimize the inclusion of tailored content specific to individual patients, with the inclusion of self-regulatory features such as goal setting to provide structured and individualized support. We believe that there is a significant rationale for involving patients in the cocreation of digital health technologies to enhance engagement, better support behavior change, and improve overall surgical outcomes for patients.

## Appendix

Multimedia Appendix 1 Supplementary data, including meta-ethnographic data synthesis.

## Abbreviations

CASP: Critical Appraisal Skills Programme
iCBT: internet-delivered cognitive behavioral therapy
PRISMA: Preferred Reporting Items for Systematic Reviews and Meta-Analyses
