# Supplementary material for: Digital and Mobile Technologies to Promote Physical Health Behavior Change and Provide Psychological Support for Patients Undergoing Elective Surgery: Meta-Ethnography and Systematic Review
Source: JMIR Mhealth Uhealth. 2020 Dec 1;8(12):e19237. doi: 10.2196/19237 (PMC7738263; doi:10.2196/19237)
Supplement: Multimedia Appendix 1 [file mhealth_v8i12e19237_app1.docx]

**Digital and mobile technologies to promote physical health behaviour change and provide psychological support for elective surgical patients: a meta-ethnography and systematic review**

**MULTIMEDIA APPENDIX 1**

**Appendices:**

Appendix A: PRISMA 2009 checklist

Appendix B: Database search terms

Appendix C: Table 1: Study characteristics

Appendix D: Table 2: Quality appraisal

Appendix E: Table 3: Determining how the studies are related: metaphors and perspectives from the included studies that lead to development of the overarching themes and sub-themes.

Appendix F: Tables 4 – 7: Qualitative data synthesis

**Appendix A: PRISMA 2009 checklist**

| **Section/topic** | **#** | **Checklist item** | **Reported on page # of submitted version** |
| --- | --- | --- | --- |
| **TITLE** | | |  |
| Title | 1 | Identify the report as a systematic review, meta-analysis, or both. | 1 |
| **ABSTRACT** | | |  |
| Structured summary | 2 | Provide a structured summary including, as applicable: background; objectives; data sources; study eligibility criteria, participants, and interventions; study appraisal and synthesis methods; results; limitations; conclusions and implications of key findings; systematic review registration number. | 1-2 |
| **INTRODUCTION** | | |  |
| Rationale | 3 | Describe the rationale for the review in the context of what is already known. | 2-3 |
| Objectives | 4 | Provide an explicit statement of questions being addressed with reference to participants, interventions, comparisons, outcomes, and study design (PICOS). | 1, 3 |
| **METHODS** | | |  |
| Protocol and registration | 5 | Indicate if a review protocol exists, if and where it can be accessed (e.g., Web address), and, if available, provide registration information including registration number. | 3 |
| Eligibility criteria | 6 | Specify study characteristics (e.g., PICOS, length of follow-up) and report characteristics (e.g., years considered, language, publication status) used as criteria for eligibility, giving rationale. | 3-4 |
| Information sources | 7 | Describe all information sources (e.g., databases with dates of coverage, contact with study authors to identify additional studies) in the search and date last searched. | 4 |
| Search | 8 | Present full electronic search strategy for at least one database, including any limits used, such that it could be repeated. | 3 & Multimedia Appendix, Appendix B |
| Study selection | 9 | State the process for selecting studies (i.e., screening, eligibility, included in systematic review, and, if applicable, included in the meta-analysis). | 4 |
| Data collection process | 10 | Describe method of data extraction from reports (e.g., piloted forms, independently, in duplicate) and any processes for obtaining and confirming data from investigators. | 4 |
| Data items | 11 | List and define all variables for which data were sought (e.g., PICOS, funding sources) and any assumptions and simplifications made. | 3-4 |
| Risk of bias in individual studies | 12 | Describe methods used for assessing risk of bias of individual studies (including specification of whether this was done at the study or outcome level), and how this information is to be used in any data synthesis. | 4 |
| Summary measures | 13 | State the principal summary measures (e.g., risk ratio, difference in means). | N/A |
| Synthesis of results | 14 | Describe the methods of handling data and combining results of studies, if done, including measures of consistency (e.g., I^2^) for each meta-analysis. | N/A |
| Risk of bias across studies | 15 | Specify any assessment of risk of bias that may affect the cumulative evidence (e.g., publication bias, selective reporting within studies). | N/A |
| Additional analyses | 16 | Describe methods of additional analyses (e.g., sensitivity or subgroup analyses, meta-regression), if done, indicating which were pre-specified. | N/A |
| **RESULTS** | | |  |
| Study selection | 17 | Give numbers of studies screened, assessed for eligibility, and included in the review, with reasons for exclusions at each stage, ideally with a flow diagram. | 5-6, Figure 1 |
| Study characteristics | 18 | For each study, present characteristics for which data were extracted (e.g., study size, PICOS, follow-up period) and provide the citations. | 6 + Table 1 (Multimedia Appendix) |
| Risk of bias within studies | 19 | Present data on risk of bias of each study and, if available, any outcome level assessment (see item 12). | 6 + Table 2 (Multimedia Appendix) |
| Results of individual studies | 20 | For all outcomes considered (benefits or harms), present, for each study: (a) simple summary data for each intervention group (b) effect estimates and confidence intervals, ideally with a forest plot. | Table 1, Table 3, and Tables 4-7 (Multimedia Appendix). |
| Synthesis of results | 21 | Present results of each meta-analysis done, including confidence intervals and measures of consistency. | N/A |
| Risk of bias across studies | 22 | Present results of any assessment of risk of bias across studies (see Item 15). | N/A |
| Additional analysis | 23 | Give results of additional analyses, if done (e.g., sensitivity or subgroup analyses, meta-regression [see Item 16]). | N/A |
| **DISCUSSION** | | |  |
| Summary of evidence | 24 | Summarize the main findings including the strength of evidence for each main outcome; consider their relevance to key groups (e.g., healthcare providers, users, and policy makers). | 14-17 |
| Limitations | 25 | Discuss limitations at study and outcome level (e.g., risk of bias), and at review-level (e.g., incomplete retrieval of identified research, reporting bias). | 14 |
| Conclusions | 26 | Provide a general interpretation of the results in the context of other evidence, and implications for future research. | 17 |
| **FUNDING** | | |  |
| Funding | 27 | Describe sources of funding for the systematic review and other support (e.g., supply of data); role of funders for the systematic review. | 17 |

**Appendix B: Database search terms**

Full search = (Digital intervention) AND (Behaviour changes) AND (Mental health) AND (Surgical specialities) AND (Qualitative methodology)

**Digital intervention:**

(exp mobile health application/ or mobile phone/ OR smartphone/ OR phone.mp. OR exp text messaging/ OR digital technology.mp. OR digital device.mp. OR digital healthcare.mp. OR exp activity tracker/ OR fitbit.mp. OR videotape.mp. or videotape/ OR videotape.mp. or exp videotape/ OR wearable technology.mp. OR pedometer.mp. or pedometer/ OR exp mobile application/ or exp mobile health application/ OR social media.mp. or social media/ OR exp social network/ OR instagram.mp. OR facebook.mp. OR exp telehealth/ OR exp telerehabilitation/ OR electronic mail.mp. or e-mail/ OR ipad.mp. or tablet computer/ OR exp mobile application/ or mhealth.mp. OR video conferencing.mp. or videoconferencing/ OR smartwatch.mp. OR mobile health application/ or exp mobile application/)

**Behaviour changes:**

(physical activity.mp. OR walking.mp. OR running.mp. OR physiotheraphy.mp. OR step count.mp. OR weight loss.mp. OR weight reduction.mp. OR diet.mp. OR lifestyle.mp. OR obesity.mp. OR health behaviour.mp. OR exp Health Promotion/ OR exp Healthy Lifestyle/)

**Mental health:**

(exp Anxiety/ OR worry.mp. OR stress.mp. OR cognitive behaviour.mp. OR cognitive behavioural therapy.mp OR mindfulness.mp. OR wellbeing.mp OR mental health.mp. OR quality of life.mp. OR depression.mp OR exp Attitude to Health/)

**Surgical specialities:**

- ***Cancer:***

(cancer.mp. OR cancer patient.mp. OR exp cancer survivors/ OR cancer survivor.mp. OR cancer surgery.mp.)

- ***Bariatric:***

(bariatrics.mp. OR exp bariatric surgery/ OR bariatric surgery.mp. OR weight loss surgery.mp. OR exp obesity management/ OR obesity management.mp.)

- ***Orthopaedic:***

(orthopaedic.mp. OR knee replacement.mp. OR hip replacement.mp. OR arthoplasty.mp. OR Arthroscopy.mp. OR joint.mp. OR muscle.mp.)

**Qualitative methodology:**

(qualitative analysis/ or exp qualitative research/ OR semi structured interview/ or telephone interview/ or interview.mp. or interview/ OR focus group.mp. or information processing/ OR mixed study.mp. OR thematic analysis.mp. OR ethnography.mp.)

**Appendix C: Table 1: Study characteristics**

| Study, journal, country, and surgical type | Method of data collection | Delivery method | Population data (mean age years, SD) | Inclusion criteria | Aim | Main themes extracted from the original study |
| --- | --- | --- | --- | --- | --- | --- |
| - Shaffer et al [38] - *Psycho-Oncology* - United States - Cancer surgery | Semistructured focus group interviews | Internet-based cognitive behavioral therapy (iCBT) program | 11 cancer patients (57, insufficient data to calculate SD) | - Aged 21 years or older - Have regular internet access - Being in remission from any stage and any type of cancer - At least 1 month had passed since the completion of active treatment - Meeting DSM-IV-TE criteria for insomnia - Sleep no more than 6.5 hours per night - Self-reporting that insomnia began or worsened as a result of cancer diagnosis or treatment | To examine the cancer survivors’ qualitative feedback about internet-based cognitive behavioral treatment for insomnia | - Behavioral treatment during active cancer treatment phase - Completing behavioral treatment earlier - Align start of behavioral treatment earlier - Interest in passive interventions during active cancer treatment - Cancer-specific tailoring - Acknowledge cancer experiences are unique |
| - Phillips et al [56] - *Journal of Cancer Survivorship* - United States - Cancer surgery | Semistructured interviews and web-based questionnaire | Mobile app | Full sample, n=96. (56, SD 10.2)  Interview sample, n=28. (53, SD 10.1) | - Aged 18 years or above - Diagnosed with stage 1-3 breast cancer within the last 5 years - ≥3 months postprimary treatment - Able to read - Able to write and speak English - Own a smartphone - Have access to a computer with internet | To explore the breast cancer survivors’ preferences for mHealth^a^ physical activity interventions | - Importance of relevance to breast cancer survivors - Easy to use - Integration with wearable activity trackers - Provide sense of accomplishment - Variability in desired level of structure and personalization |
| - Nguyen et al [50] - *Supportive Care in Cancer* - Australia - Cancer surgery | Semistructured interviews | Wearable (FitbitOne, Jawbone up 24, Garmin Vivofit 2, Garmin Vivosmart, Garmin Vivoactive and Polar A300) | 14 postmenopausal breast cancer survivors (57, SD 3.8) | - Postmenopausal women diagnosed with stage 1-3 breast cancer - Completed their primary treatment at least 6 months ago - Residing in Victoria, Australia - Able to speak and write fluently in English - Have daily access to a handheld device or personal computer or internet | To explore the acceptability and usability of wearable technology activity trackers among postmenopausal breast cancer survivors | - Trackers’ increased self-awareness and motivation - Breast cancer survivors’ confidence and comfort with wearable technology - Preferred and disliked features of WAT^b^ - Concerns related to the disease - Peer support and doctor monitoring |
| - Alberts et al [40] - *Supportive Care in Cancer* - United States - Cancer surgery | Semistructured interviews | Internet-based, *Wellbeing after cancer* iCBT course | 13 cancer survivors and 10 providers (53, SD 8.2) | - Have partial or complete remission from any type of cancer as long as ≥1 and ≤8 months had passed since active treatment | To explore patient and provider perceptions of internet-delivered cognitive behavioral therapy for recent cancer survivors | - Aspects of the program participants liked: “I’m not alone,” design and organization, length and pace, flexibility, privacy and fit, relationship with therapist, course content and associated changes - Aspects of the program participants disliked and/or would like to be improved: additional information on side effects and conditions, increased flexibility, break up lessons, more directive, and difficult to identify dislikes - Barriers to completing the program: finding the time and physical and mental barriers - Program strengths: accessibility, program features, support after treatment, and utility in current work |
| - Gell et al [41] - *Supportive Care in Cancer* - United States - Cancer surgery | Semistructured interviews | Combination of wearable technology (Fitbit) and text messaging | 19 cancer survivors (56, SD 10.9) | - A history of a cancer diagnosis - Access to a personal cell phone - Expected completion of the oncology rehabilitation program - Able to speak and write in English | To examine female cancer survivors’ perspectives on remote monitoring and communication to support independent physical activity maintenance | - Accountability to a remote partner: silent partner, physical reminder, watchful eye, encouragement fostering accountability - Plan Bs, planning for barriers: overcoming interference, accommodations, problem-solving - The habit cycle: social support, positive health effects, reinforcement, tenuous transition - Convenience through technology: accessible, ease, informative - Reclaiming the health ownership following a cancer diagnosis: overcoming fatigue, emotional and physical aspects of health, renewing social connections |
| - Kokts-Porietis et al [47] - *Supportive Care in Cancer* - Canada - Cancer surgery | Semistructured interviews | Activity tracker (Polar A360) | 6 breast cancer survivors (58, SD 9) | - 18-75 years of age - Diagnosed with stage 1-3c breast cancer - Physically inactive (<10,000 steps per day and <60 min of moderate-vigorous intensity physical activity per week) - Completed all adjuvant treatment except for hormonal therapy - Resident of Calgary, Canada | To gain breast cancer survivors’ perspectives on participation in a home-based physical activity intervention and the factors that contributed to their acceptance and adherence to physical activity | - Study environment: arch versus fear of failure, power of results, and reminders of cancer, and moving beyond - Influence of people: personal relationships and self-motivation - Wearable technology: objective insights into health and disconnect of person and technology |
| - Webb et al [44] - *Public Health* - United Kingdom - Cancer surgery | Questionnaire and semi structured interviews | Internet-based, “Move More Pack” | 17 cancer patients (54, SD 11.1) | - Aged 18 years or above - Having mixed tumor sites, cancer stages, and levels of physical activity | To understand if age, gender, cancer status, or tumor site influences use of an intervention supported by internet tools to improve physical activity in cancer survivors in the United Kingdom | - Capitalizing the teachable moment - Already moving - I am highly active - Physical activity is not for everybody |
| - Lally et al [42] - *Oncology Nursing Forum* - United States - Cancer surgery | Structured interview for synchronous online focus groups | Web-based, online discussion forums | 23 breast cancer survivors (59, SD 11.6) | - First diagnosed with stage 0-3a breast cancer in one month to 10 years - Living in rural counties designated as 6 to 9 by the US Department of Agriculture Rural-Urban Community Area Codes or living in a zip code designated as 10 | To obtain rural breast cancer survivors’ perceptions of the quality and usability of web-based distress self-management program | - Quality: time, relevance and trustworthy - Usability: navigable and comfortable |
| - Zhu et al [55] - *JMIR mHealth and uHealth* - China - Cancer surgery | Semistructured interviews | Mobile app, e-support program | 13 breast cancer patients (36, SD 14.8) | - Had commenced chemotherapy at the study sites after diagnosis of breast cancer - Able to access the internet via a mobile phone - Able to read and write Mandarin | To explore the participants’ perception of Breast Cancer e-support program, its strengths and weaknesses, and suggestions to improve the program | - Benefits of breast cancer e-support program: enhanced knowledge, improved confidence level, improved emotional well-being, received advices from experts, easy to use, easily accessible and convenient - Challenges to engagement: physical or psychological health status, stigma with breast cancer, instability of the app - Suggested improvement: design improvement, interesting, plain, and practical content, the information being updated more often, quicker responses to women’s questions Future direction: breast cancer e-support program as routine care, open to caregivers and applied to other patients with cancer |
| - Hardcastle et al [51] - *PLOS ONE* - Australia - Cancer surgery | Semi structured interviews | Wearable technology (Fitbit Alta, Garmin Vivofit 2, Garmin Vivosmart, Polar Loop 2, and Polar A300) | 20 patients with cancer  (63, SD 13) | - Had completed active treatment for cancer within the preceding 5 years and deemed to be in remission - Insufficiently physically active - Resided in a regional and remote areas of Western Australia - Had daily access to a handheld device or personal computer and internet | To investigate the acceptability of and preference for wearable activity trackers among nonmetropolitan cancer survivors | - Increasing self-awareness of PA^c^ and SB^d^ - Prompts and feedback - Accuracy and registry of activities - WAT preferences and features: appearance and functionality |
| - Rosenberg et al [43] - *American Medical Informatics Association* - United States - Cancer surgery | Semi structured in-depth interview and baseline survey | Wearable technology (Fitbit) | 26 patients with prostate cancer (71, SD 9.7) | - Having prostate cancer - Able to stand - Able to walk one block - Able to speak and read English | To investigate the acceptability of Fitbit for physical activity tracking within clinical care among men with prostate cancer | - Wearability - Ease of using technology - Value in using - Barriers to use - Priority Fitbit features - Attitudes toward integrating Fitbit with care |
| - Puszkiewicz et al [45] - *JMIR Cancer* - United Kingdom - Cancer surgery | Semi structured interviews | Mobile app | 11 patients with cancer (45, SD 9.4) | - Aged 18 years or above - Diagnosis of breast, prostate, or colorectal cancer - Have finished primary curative treatment - Own an iPhone | To assess the cancer survivors’ experiences of using a publicly available physical activity mobile app | - Barriers to PA - Receiving advice about PA from reliable sources - Tailoring the app to one’s lifestyle - Receiving social support from other cancer survivors |
| - Clarke et al [46] - *BMC Health Services Research* - United Kingdom - Cancer surgery | Semi structured interviews | Web-based assessment, prostate cancer web-based holistic needs assessments | 16 patients with prostate cancer (age range: 61-85 years, SD NR) | - Have a prostate cancer diagnosis - Able to communicate in English - Under the care of the participating practices | To identify perceived barriers and motivators to implementation and continued use of cancer-specific holistic need assessment | - Perceived consequences and optimism - Perceived value and impact on care (beliefs about consequences, motivation, optimism) |
| - Lee et al [54] - *Journal of Rehabilitation Research and Development* - South Korea - Orthopedic surgery | Open-ended questionnaire and physical assessment surveys | Virtual reality–based rehabilitation | 25 orthopedic patients (367, SD 14.8) | - More than 4 weeks had passed since the operation - Can stand independently - Have a normal cognition (mini-mental state examination score>25) | To explore the perspectives of knee surgery patients regarding virtual reality–based rehabilitation | - Usability: difficulty, enjoyment, concentration, pain and unity - Engagement: goals and feedback |
| - Kairy et al [48] - *International Journal of Environmental Research and Public Health* - Canada - Orthopedic surgery | Semi structured interviews | Telerehabilitation | 5 orthopedic patients (61, SD 9.9) | - Previously received physiotherapy services in the community | To explore patients’ perceptions regarding tele-rehabilitation services received after total knee replacement | - Improving access to services - Developing a bond with their therapist while maintaining a sense of personal space - Complementing tele-rehabilitation with in-person visits - Providing standardized yet tailored and challenging exercise programs using tele-rehabilitation - Perceived ease-of-use tele-rehabilitation equipment - Achieving an ongoing sense of support |
| - Argent et al [52] - *Sensors* - Ireland - Orthopedic surgery | Semistructured interview, System Usability scale (SUS), mobile app rating scale (uMARS) | Wearable technology (type NR^e^) | 15 orthopedic patients  (NR) | - Living within 30 km of the hospital - Have no history of cognitive dysfunction - No difficulty understanding English | To evaluate an exemplar sensory-based biofeedback system, investigating the feasibility, usability, perceived impact, and user experience of using the platform | - Usability: functionality and user experience - Perceived impact: support and motivation, improving adherence, and increasing confidence - Suggestions for refinements or additional features**:** additional exercises, measurement of the joint angle, quality score, improved graphical interfaces and gamification |
| - Das and Faxvaag [53] - *Interactive Journal of Medical Research* - Norway - Bariatric surgery | In-depth semistructured interviews | Web-based, online discussion forums | 7 bariatric patients  (60, SD 9.3) | - Age 18 years or above - Basic proficiency in the Norwegian language - Enrollment in a bariatric weight loss program at the hospital | To explore how individuals undergoing bariatric surgery used the moderated discussion forum, and to better understand what influenced their participation | - Informational support, advice, and guidance - Social support and networking among peers - Concerns regarding self-disclosure |
| - Atwood et al [49] - *Health Communication* - Canada - Bariatric surgery | Online forums | Web-based, online discussion forums | 1412 messages, preop^f^ (n=822) and postop^g^ (n=590) | - Availability of active forums with daily message posts - Availability of a large number of members and message posts - Public accessibility - The willingness and permission of the website authority or administrator to utilize message posts for this study | To examine the types of social support found on online bariatric surgery support forums, and the frequency with which these support types are exchanged among forum members | - Informational support**:** providing factual information, suggestions or advice, alternative perspectives on a situation, and referrals to additional sources of help - Emotional support: encouragement, sympathy and validation - Network support: presence, access, companions - Esteem and tangible support: compliment and willingness to help |

^a^mHealth: mobile health.

^b^WAT: wearable activity tracker.

^c^PA: physical activity.

^d^SB: sedentary behavior.

^e^NR: not reported.

^f^preop: preoperative period (before surgery).

^g^postop: postoperative (after surgery).

**Appendix D: Table 2: Quality appraisal**

Table 2: Quality appraisal

| **Author & Year** | **Critical Appraisal Skills Tool Screening Questions** | | | | | | | | | | **Comments** |
| --- | --- | --- | --- | --- | --- | --- | --- | --- | --- | --- | --- |
|  | **1. Was there a clear statement of the aims of the research?** | **2. Is qualitative methodology appropriate?** | **3. Was the research design appropriate to address the aims of the research?** | **4. Was the recruitment strategy appropriate to the aims of the research?** | **5. Was the data collected in a way that addressed the research issue?** | **6. Has the relationship between researcher and participants been adequately considered?** | **7. Have ethical issues been taken into consideration?** | **8. Was the data analysis sufficiently rigorous?** | **9. Is there a clear statement of findings?** | **10. How valuable is the research?** |  |
|  | *Yes/No/Can’t tell* | | | | | | | | | |  |
| Shaffer *et al.* (2019)[35] | Yes | Yes | Yes | Yes | Yes | Yes | Yes | Yes | Yes | Yes |  |
| Philips *et al.* (2018)[54] | Yes | Yes | Yes | Yes | Yes | Yes | Yes | Yes | Yes | Yes |  |
| Nguyen *et al.* (2017)[48] | Yes | Yes | Yes | Yes | Yes | Yes | Yes | Yes | Yes | Can’t tell | **Q10** – Unclear presentation of how the findings could be transferred to other populations or other ways the research may be used. |
| Alberts *et al.* (2017)[37] | Yes | Yes | Yes | Yes | Yes | Yes | Yes | Yes | Yes | Yes |  |
| Gell *et al.* (2019)[38] | Yes | Yes | Yes | No | Yes | Yes | Yes | Can’t tell | Yes | Yes | **Q4** – No explanation why the selected participants were the most appropriate.  **Q8** – Small sample size, therefore the findings cannot be generalised. |
| Kokts-Porietis *et al.* (2019)[45] | Yes | Yes | Yes | No | Yes | Yes | Yes | Yes | Yes | Yes | **Q4** – No discussion around why some participants chose not to take part in the qualitative study. |
| Webb *et al.* (2019)[41] | Yes | Yes | Yes | Yes | Yes | Yes | Can’t tell | Yes | Yes | Can’t tell | **Q7** – Lacking details of how the research was explained to participants.  **Q10** – No specification on new areas where research is necessary. |
| Lally *et al.* (2018)[39] | Yes | Yes | Yes | Yes | Yes | Yes | No | Yes | Yes | Can’t tell | **Q7** – No statement around approval of the ethics committee to assess whether ethical standards were maintained.  **Q10** – No report of new areas where research is necessary and whether the findings can be transferred to other populations. |
| Zhu *et al.*  (2018)[53] | Yes | Yes | Yes | Can’t tell | Yes | Yes | No | Can’t tell | Yes | Yes | **Q4** – Unclear discussion around the recruitment.  **Q7** - No statement around approval of the ethics committee.  **Q8** – Only two quotations were reported for each subthemes, so there is insufficient data presentation. |
| Hardcastle *et al.* (2016)[49] | Yes | Yes | Yes | Yes | Yes | Yes | No | Yes | Yes | Yes | **Q7** - No statement around approval of the ethics committee to assess whether ethical standards were maintained. |
| Rosenberg *et al.* (2017)[40] | Yes | Yes | Yes | Yes | Yes | Yes | No | Yes | Yes | Yes | **Q7** – No statement around approval of the ethics committee or whether the research was explained to the participants for the reader to assess whether ethical standards were maintained. |
| Puszkiewicz *et al.* (2016)[42] | Yes | Yes | Yes | Yes | Yes | Can’t tell | Yes | Yes | Yes | Yes | **Q6** - Unclear if the researcher critically examined their own role, potential bias and influence during formulation of the research question. |
| Clarke *et al.* (2019)[43] | Yes | Yes | Yes | Yes | Yes | Yes | Yes | Can’t tell | Yes | Yes | **Q8** – Thematic analysis is used, however it is unclear how the categories/themes were derived from the data. |
| Lee *et al.* (2016)[52] | Yes | Yes | Yes | Yes | Yes | Yes | No | Yes | Yes | Yes | **Q7** - No statement around approval of the ethics committee for the reader to assess whether ethical standards were maintained.  **Q8** – Insufficient data presentation to support the findings (small sample size).  **Q9** – Inadequate discussion around the qualitative analysis. |
| Kairy *et al.* (2013)[46] | Yes | Yes | Yes | Yes | Yes | Can’ tell | Yes | Can’t tell | Yes | Can’t tell | **Q6** – Unclear if the researcher critically examined their own role, potential bias and influence during formulation of the research question.  **Q7** – The researchers have stated the study was approved by the appropriate ethics review board, but the name of the committee was not specified.  **Q8** – Small sample size, therefore the findings cannot be generalised.  **Q10** - The researchers have not reported any new areas where research is necessary and whether or how the findings are transferable. |
| Argent *et al.* (2019)[50] | Yes | Yes | Yes | Yes | Yes | Yes | Yes | Yes | Yes | Yes |  |
| Das *et al.* (2014)[51] | Yes | Yes | Yes | Yes | Yes | Yes | Yes | Can’t tell | Yes | Can’t tell | **Q8** – Small sample size, therefore findings cannot be generalised.  **Q10** – The study is limited to one discussion forum for bariatric surgery patients, and the results cannot be transferred to other patient population or other health forums. |
| Atwood *et al.* (2018)[47] | Yes | Yes | Yes | No | Yes | Yes | No | Can’t tell | Yes | Yes | **Q4** – No information regarding the selection or the inclusion criteria of the participants.  **Q7** - No statement around approval of the ethics committee for the reader to assess whether ethical standards were maintained.  **Q8** – Messages analysed are from one publically available online support forum for individuals who have undergone RYG, thus the findings cannot be generalised. |

**Appendix E: Table 3: Determining how the studies are related: metaphors and perspectives from the included studies that lead to development of the overarching themes and sub-themes.**

| Study | Providing motivational support | Addressing patient engagement | Facilitating peer networking | Meeting individualized patient needs |
| --- | --- | --- | --- | --- |
| Shaffer et al [38] | — | — | — | When to offer DHT^a^ intervention vs when not to offer, what to include vs what not to include, appropriate tailoring |
| Phillips et al [56] | Provide a sense of accomplishment, personal motivation, goal setting, reward behaviors | Easy to use, simplistic design | — | Importance of tailoring information (relevance to breast cancer survivors), content specificity to intended patient cohort, integration of DHT with wearable technologies, structure and personalization of DHT |
| Nguyen et al [50] | Self-awareness and motivation, personal goal setting, value of HCP monitoring to drive motivation | Importance of building confidence with technology, comfort affects engagement with wearable DHTs | Peer support, helping each other | Tailoring to concerns relating to a specific disease or treatment, preferred and disliked features |
| Alberts et al [40] | Personal support, “starting to realize” motivation, awareness, value of HCP monitoring to drive motivation | Simplicity, important to feel relaxed while using, desire to engage, recognized accessibility and availability of DHTs | Peer support, “I’m not alone” in experiences, shared coping techniques and support | — |
| Gell et al [41] | Reclaiming health ownership, source of personal support, support to overcome challenges and stay on track, creating a habit cycle | Convenience of use | — | — |
| Kokts*-*Porietis et al [47] | Self-motivation, personal responsibility, but caution in case of “overmotivating” turning into technology as a source of judgement (disconnect between person and tech) | Offering objective insights into personal health, usability | — | Improvements, suggestions to improve study environment and DHT intervention |
| Webb et al [44] | Personal motivation and responsibility to keep moving, capitalizing on teachable moments, motivation and responsibility, awareness of responsibility of not exercising | — | — | — |
| Lally et al [42] | — | Easy to navigate, search tools, comfort and ease with the web-based discussion tool | Relevant information shared from peer support | When to start, reflections about initiation and expected engagement, tailoring to the specific needs of patient cohort |
| Zhu et al [55] | Supported through readily accessible information from HCP^b^, tailored expert advice to support | Stigma of breast cancer can affect engagement—not using the app as it reminds them of the disease, instability of the app (app failure and inaccurate measurements of activity resulted in lack of engagement, users ‘gave up’ with logins) | Improved confidence and well-being | Specific information for disease and surgery type, keep learning and education focused for patient cohort, individually tailoring content and amount of information (not too much if unwell), suggested improvements |
| Hardcastle et al [51] | Motivation from DHT prompts and feedback, personal motivation and responsibility | Recognized inaccuracies with registering activities, not trustworthy. | — | Preferences and features, preferred style and acceptability |
| Rosenberg et al [43] | Finding value in moving, personal motivation, recognizing benefits of tracking, attitude to integrating DHT interventions in care, support through data-sharing. | Ease of using DHT, simple, ease to synchronize, wearability of activity trackers, recognized barriers to use (not accurately measuring activity), lack of trust in measurements or total values | DHT intervention features enabling peer support and motivation | — |
| Puszkiewicz et al [45] | Supporting participant motivation to seek information, weekly reminders to keep participants motivated, receiving advice from reliable sources | — | Social support from other cancer survivors, peer support | Tailoring to specific disease states, tailor the app to individuals’ lifestyle |
| Clarke et al [46] | Recognized benefits through connectivity with HCPs, supported information-seeking behaviors | — | — | — |
| Lee et al [54] | Engagement as motivation in goal setting, motivation from feedback | Avoidance of complex/difficult DHTs, desire for enjoyment with use | — | — |
| Kairy et al [48] | Achieving a sense of support through connectivity and information provision, developing a bond with their therapist | Standardized, easy, simple to use, DHT improving access to services and advice | — | Integration into care pathways, complementing DHT intervention with in-person visits |
| Argent et al [52] | Perceived impact through improved adherence and motivation to exercise | Simplicity, easy to use | — | Refinements, suggestions for improvement and added features |
| Das and Faxvaag. [53] | — | — | Social support and networking, providing advice/giving advice to others in the same position, encouragement, sharing experiences, purpose | — |
| Atwood et al [49] | Emotional support from others like you to keep you motivated, validation and sympathy of experience | — | Informational support from peers, dietary advice, advice on personal experiences, medication advice, network support, emotional support, sharing the experience (“journey”), building self-esteem, willingness to help others going through the same, validation and sympathy of experience | — |

^a^DHT: digital health technology.

^b^HCP: health care professional.

**Appendix F: Tables 4 - 7: Qualitative data synthesis**

Table 4: Theme 1: Providing motivation and support

| **Synthesised themes (third order constructs)** | **Sub-themes** | **Second order constructs: the authors interpretations of the original findings** | **First Order constructs: examples of direct quotations from the participants of the study** |
| --- | --- | --- | --- |
| **PROVIDING MOTIVATIONAL**  **SUPPORT** | ***Personal responsibility***  ***and motivation*** | Being accountable to text messages and Fitbit motivated the patients to be physically active and alternative exercise options identified through the interaction with health coach, which helped patients to increase confidence. (Gell *et al.*, 2019) | *“I did feel accountable because of the text that came in. So, I think that was definitely a motivator for me…”* |
|  |  |  | *“I always had a back-up plan…the coach – when we discussed at the beginning of the program, she was asking me, “what other things would you do if you could not do that exact exercise. So, that’s when we discussed, oh I’ve got tapes or if I get bored with the treadmill, or if I want to do something else, so I got the exercise tapes that I can do.”* |
|  |  |  | *“I know it’s good for me, makes me feel better and it certainly mentally makes me feel better, I’m much happier when I exercise.”* |
|  |  | Cancer survivors liked the idea of an app keeping them accountable and oriented towards the goal. Being rewarded via positive feedback and encouraging messages also provided a sense of accomplishment. (Phillips *et al.*, 2019) | “*Seeing your progress, I think is very important. Seeing measurable progress, whether it’s in calories burned, or minutes, or meeting a percentage of your goal.”* |
|  |  |  | *“I like the idea of positive feedback wherever the source. I think that’s super huge for anyone and especially for survivors because after you have cancer you are always a little bit like how is everything in there?”* |
|  |  | Clear and achievable goal, represented by the specific number of steps was the most significant motivation for the participants. (Nguyen *et al.*, 2016) | *“If you get to say 8000 [steps] in a day, you’re motivated to do those extra 2000 because you’re so close. It’s like “Why would I stop now?” I might as well keep going.”* |
|  |  | The program provided a sense of support and improved the patient’s ability to cope with difficulties, such as fear of cancer recurrence. (Alberts *et al.*, 2018) | *“And in all fairness, I will tell you right now, my anxiety levels are through the roof. And I am using every one of your magical steps in the program, because… I have found another lump…So you know again I have tools now, to keep me a little calmer.”* |
|  |  | Since adhering to self-tracking increased the physical activity and motivation, some patients expressed falling short of physical activity goals perceived as failing to achieve one’s best self. (Kokts-Porietis *et al.*, 2019) | *“Helps with your mental capacity of how to take it and control it […] I think the more active you are, the less you think about ‘Am I gonna live, am I gonna die?’”* |
|  |  |  | *“Started to enjoy exercise again and felt better […] mental health wise.”* |
|  |  |  | *“for now, I don’t wanna be judged or evaluated or anything else…and then that will change…It’s just a case of you get tired of [judgement]”* |
|  |  | Cancer survivors who are in the early stage of the disease found the intervention more useful in comparison to those experiencing particularly harsh consequences of treatment. (Webb *et al.*, 2019) | *“[The Move More Pack] gave me ideas about different activities I could participate in and where and how to access them. It is a good reminder…and you can keep a record of personal activity. I like the goal-setting and the tips.”* |
|  |  |  | *“When I was going through my chemo I tried to keep as active as possible and I could see even that was helping me. And then radiotherapy, because people said, oh it makes you so, so tired, and yes it did but if you can push through that tiredness it makes you feel so much better.”* |
|  |  |  | *“My health is not good… I have been diagnosed with polymyalgia which makes me tired and in constant pain. My involvement in any exercise is practically nil.”* |
|  |  | Wearable activity trackers provided continuous self-monitoring and personalised feedback, thus increased self-awareness towards physical activity and sedentary behaviour were observed amongst the patients. (Hardcastle *et al.*, 2018) | *“they motivated me…made me very aware of how much I’m moving and that I need to move more”* |
|  |  |  | *“Quantitative data are good because you’re reminded that you’re not meeting your targets…if set at 10,000 and you get 7,000 for a couple of days then maybe you could get direct messages, bit of a psych talk…automated but make it personalised, you missed your target for a few days rather than saying Move!”* |
|  |  | Participants expressed increased activity levels with Fitbit use and also improved motivation to achieve daily step counts. (Rosenberg *et al.*, 2017) | *“I think I like to make sure I'm doing some minimal amount of activity. And it’s kind of fun to see what you ‘ve been doing, how many steps you’ve done, how many miles you’ve gone.”* |
|  |  |  | *“I like it. I mean, it gives me weekly updates. Every now and then we‘ll challenge our daughters because the whole family has one now. We bought them for them, too. So we ‘ll do a challenge every now and then, and I'll try to kick butt.”* |
|  |  | Having the specific goal in the game motivated the participants to achieve their daily physical activity levels. (Lee *et al.*, 2016) | *“Having the specific goal of putting the ball in the hole made me interested in the game.”* |
|  |  | The use of wearable sensors offered a biofeedback in support of the home-exercise programme thus increasing the adherence to the rehabilitation programme, following the orthopaedic surgery. (Argent *et al.*, 2019) | *“It kept me doing physio when I might not have done it at home, especially with various things that have been happening at home. So it kept me doing physio and made sure I did it every day.”* |
|  | ***Connectivity to healthcare professionals*** | Involvement of the healthcare professionals, such as answering queries and providing timely feedback upon patients’ needs optimised efficacy on information delivery and reinforced the engagement with the application. (Zhu *et al.*, 2018) | *“When I faced with something I didn’t know, I was so anxious. But my doctor was very busy and he had no time to communicate with me. After I joined your program, I could ask questions through the app regarding my medical condition. I could upload the lab results through your program. Then I received corresponding advice from experts. I felt followed up. When I knew more about my medical condition, I felt more likely to gain control of my life.”* |
|  |  |  | *“I have no difficulty in using the app. I live far away from the hospital and I have no doctor close to me. When I had questions about my medical condition, I could not find the answer in the internet. Then I asked questions through the app. Aha, the professor or expert responded. Sometimes they gave me quick feedback. Sometimes, they answered my questions the next day. Yes, we can also make our judgement, but we are not sure at that time. The response from the expert provided me the direction. I believe this is the strength of the app.”* |
|  |  | Participants expressed willingness to share their Fitbit data with healthcare providers to be able to discuss their activity levels and receive advice. (Rosenberg *et al.*, 2017) | *“That would be fine with me. I think if it would help a physician or someone understands how you ‘re doing, there would be no problem with that.”* |
|  |  | The online prostate cancer-specific holistic needs assessment facilitated an opportunity to raise unmet needs that were beyond routine clinical questioning and comforted patients by extra focus from healthcare professionals. (Clarke *et al.*, 2019) | *“…it was a reinforcement of the things that I already had available to me and it was a comfort to know …that I’d got reliable medical people …available to help me if I needed it…”* |
|  |  | The participants expressed the information on upcoming steps and appointments provided by telerehabilitation technical support team was clear and the ongoing communication helped the development of relationship and trust between the patient and therapist. (Kairy *et al.*, 2013) | *“They had told me that it would be this way (…). So being advised, you know, you’re ok. (…) This way, being advised of the date, that the beginning of the treatments will be on such and such a date. And having the little handouts that said which exercises to do, well then ultimately, it was positive regardless. We say well we’re heading in, in the right direction… to recuperate.”* |
|  |  |  | *“…we talked about fishing, we talked about hunting, (…) we talked about skiing, hum, of all sorts of things, while I was doing my exercises, we talked about anything and we always had something to say. I think that she knew my whole life (laughter) (…)”* |
|  |  | The online forum provided an ease for patients who experience difficulties in making direct contact with the professionals due to personal barriers. (Das and Faxvaag, 2014) | *“I think it is very positive that you can ask questions that are conveyed to a dietician or a doctor because I must admit that picking up the phone and asking someone is very challenging. That barrier–I think it is difficult. What if it’s only me? How ridiculous! You get that feeling. Then, it is easier to write online.”* |

Table 5: Theme 2: Addressing patient engagement

| **Synthesised themes (third order constructs)** | **Sub-themes** | **Second order constructs: the authors interpretations of the original findings** | **First Order constructs: examples of direct quotations from the participants of the study** |
| --- | --- | --- | --- |
| **ADDRESSING PATIENT ENGAGEMENT** | ***Usability*** | Although most patients found Fitbit easy to wear and comfortable, variation identified regarding perceived ease of use. While syncing the device, some patients experienced technological barriers and some found it easy to use. (Rosenberg *et al.*, 2017) | *“Once I put it on in the morning I was totally unaware of its presence on my body or in my pocket*.” |
|  |  |  | *“Every day or two I sync it. I download it to Fitbit application for my iPhone and so every day or two I sync it. And then I just sort of look at the information there … It’s very easy. The Fitbit application is very — I think the term is user friendly.”* |
|  |  |  | *“Largely, I'm not wearing it because it doesn‘t interact with my computer very easily…why bother? I just go use my manual step counter.”* |
|  |  | Patients liked the design and organisation of the programme, which expressed as simple and straightforward. (Alberts *et al.*, 2018) | *“Well it was very simple. It was straightforward. It wasn’t complicated…like going through chemo you have kind of a brain scramble and… just the simplest things you can’t wrap your brain around sometimes.”* |
|  |  | Patients expressed the simplicity is the key for the adherent engagement with the application. (Phillips *et al.*, 2019) | *“I would say the most important thing is the ease of use, the simplicity of it because if it’s cumbersome I will not use it.”* |
|  |  | Self-monitoring through the wearable allowed patients to track their step counts and increased convenience through technology. (Gell *et al.*, 2019) | *“It’s very easy to use… It’s something fun for me to do, to look at where my day ended yesterday and whether I was on target or needed to refocus a bit.”* |
|  |  | The correct balance between the task difficulty and personal skill levels identified as prerequisite for the continuous engagement with the intervention. (Lee *et al.*, 2016) | “*The level of challenge was suitable for me, so I got a good score.”* |
|  |  |  | *“I found it difficult to understand how to perform this exercise.”* |
|  |  |  | *“I sensed a lack of unity between my movement and that of the virtual character, so I got a bad score; this made me lose interest in the game.”* |
|  |  | Patients reflected a positive reinforcement of self-tracking with ease of using the wearable technology. (Kokts-Porietis *et al.*, 2018) | *“set goals, like mid-week if I wanna hit 150 [minutes] I should be at half that […] and the application is on my phone and I can see what I’ve done […] so it’s really easy to track how well you’re doing or how well you’re not doing.”* |
|  |  | Some patients encountered technical difficulties while operating the tracker and application, whereas some expressed confidence and comfort of using the intervention. (Nguyen *et al.*, 2017) | *“Once I understood how it worked, it was easy, you know you hit sync on this, open the app and little whirly twirly things happened and it spat out the findings so”* |
|  |  |  | *“I am a bit old school I don’t think the experience [using the tracker] would make me necessarily go out and buy one. If I felt I was reasonably active doing what I do, in my normal daily activities, I would probably be happy with that, but then I tend to ignore technology if I can.”* |
|  |  | The usage of the program reduced due to some patients experiencing technological difficulties and stigma with breast cancer which acted as a perpetual reminder of their disease. (Zhu *et al.*, 2018) | *“If I told my friends that I had breast cancer, they would reject me. I had such experience...They perceived me as a different person. How can I have the courage to tell people about my disease? I do not want to touch the topic of “breast cancer”. I’ve tried to put it behind me...Using this program, reading and chatting, it constantly reminds me of my illness. I need to be done with it.”* |
|  |  |  | *The app sometimes was unstable. It didn’t work when I tried to open it. I contacted with someone in the hospital and reinstalled the app. Then I could log in. However, after a periods of time, I couldn’t open the app again. Finally I gave up using your program. I haven’t log in for the recent month.”* |
|  |  | Regardless the perceived literacy with technology, most patients found the intervention easy to use. (Argent *et al.*, 2019) | *“Initially I said to you I wasn’t very computer literate but it’s very simple to use. Once you do it once or twice you can do it with your eyes closed essentially.”* |
|  |  | The visual instructions of the application helped patients to feel confident about how to perform the exercises correctly. (Puszkiewicz *et al.*, 2016) | *“[The visuals] were really good because [they] showed you how to do everything and you felt confident that you are doing it right.”* |
|  |  | Patients found the programme easy to navigate and comfortable, meeting their expectations and needs. (Lally *et al.*, 2018) | *“It just took a moment to log-in and navigation was speedy!”* |
|  |  |  | *“I like that the color scheme was NOT pink!”* |
|  |  | Patients expressed positive feelings about ease-of-use and usefulness of the program. (Kairy *et al.*, 2013) | *“I installed the things I needed. Like that, all my bicycle, and hum... my step. I installed that and it went well. Look, it took 2 min.”* |
|  | ***Reliability*** | The instability of the application reduced the patient’s reliability to continue engage with the intervention. (Zhu *et al.*, 2018) | *“The app sometimes was unstable. It didn’t work when I tried to open it. I contacted with someone in the hospital and reinstalled the app. Then I could log in. However, after a periods of time, I couldn’t open the app again. Finally I gave up using your program. I haven’t log in for the recent month.”* |
|  |  | The inaccuracy of the statistics and inability of recording the other activities led to disappointment in patients. (Hardcastle *et al.*, 2018) | *“My hand was moving for a couple of minutes, it thought I was running.”* |
|  |  |  | *“It doesn’t take into account other exercise like swimming”* |
|  |  | The activity tracker failed to capture important daily living activities which identified as a barrier in terms of engagement with the intervention. (Rosenberg *et al.*, 2017) | *“So I'll give you a case. I filled my laundry, and it’s logged I walked 2,000 steps. I did not walk 2,000 steps.”* |
|  |  |  | *“I guess the only surprise was that it seemed to register less activity than I felt I actually did because it was only measuring steps, and I was doing more than steps. I was lifting. I was bending. I was twisting. I was doing all that other sort of stuff.”* |
|  | ***Accessibility*** | The elimination of the transportation time mentioned as predominant benefit of the tele-rehabilitation for both the patient and therapist. (Kairy *et al.*, 2013) | *“I really like it (tele-rehabilitation). I found it fantastic…you know, just the fact of not having to travel when we are in pain (…) I adored it.”* |
|  |  | The availability of the program to the individuals in rural areas reduced the clinic visits. (Alberts *et al.*, 2018) | *“Well, definitely the availability of it to anybody, no matter where you live. I know we work with a lot of rural people and after they’re done here, they don’t want to travel for more therapy or whatever, so something that they can do at home.”* |

Table 6: Theme 3: Facilitating peer networking

| **Synthesised themes (third order constructs)** | **Sub-themes** | **Second Order constructs: the authors interpretations of the original findings** | **First Order constructs: examples of direct quotations from the participants of the study** |
| --- | --- | --- | --- |
| **FACILITATING PEER NETWORKING** | ***Educational development*** | The discussion forum allowed patients to open up and share the challenges of losing weight and motivational difficulties which promoted the acknowledgement and social support. (Das *et al.*, 2014) | *“I think it is more enjoyable to write a “diary” that everyone can read and comment on. I like to get feedback on how I do things, what I eat, and thoughts that I have about the surgery and about life after the operation, so here comes a little of everything…Hope you will read and comment.”* |
|  |  |  | *“It is actually the support and the approval regarding what you are doing, feedback regarding whether it is right, and feedback regarding insecurities.”* |
|  |  | The informational support intended to provide recommendations regarding the dietary guidelines and physical activity in order to facilitate the post-surgery weight lost in bariatric patients. (Atwood *et al.*, 2018) | *“[Product name]… this is odorless and tasteless and does not clump. You can add it to hot or cold… or just sprinkle over your food. One tablespoon equals a scoop of Whey and has 30 grams of protein. It is approved by [Medical Association] and has 96% absorption…”* |
|  |  |  | *“… You may want to pick up a pill crusher and a pill splitter in the drug store. The large pills such as calcium citrate, I had to crush and mix with drink in order to take them…”* |
|  | ***Connecting with others*** | The statements of encouragement aimed to provide patients confidence regarding the progress of adhering to dietary and physical exercise to increase the surgery outcome. (Atwood, 2018) | *“It does seem like you are hitting a lot of bumps in the road. Keep your sense of humour. It’ll all be worth it in the end.”* |
|  |  |  | *”I would be happy to make this journey with you…I would love to be your buddy.”* |
|  |  |  | *“… If you have any questions about recipes, diet, or exercise, I am an open book…”* |
|  |  | The social support required to build a sense of community among the patients who could share the same experiences in order to support each other to achieve targeted physical activity goals. (Puszkiewicz, 2016) | *“If you are looking at the issues of cancer survivorship, I think personally that for cancer survivors it would be quite nice to link up with other people and build that community.”* |
|  |  |  | *“You do need that bit of motivation from other people. It’s all about motivation when it comes to exercise […]. When you feel low and can’t be bothered to go for a walk, maybe someone else saying ‘go on, get up and do it, you can do it’ would motivate you.”* |
|  |  | Peer support encouraged and motivated patients to carry out the exercise with the individuals having the same medical condition with the same activity trackers. (Nguyen, 2017) | *“In this day of social media I have a lot of groups of friends that are Fitbit people and they have their own groups and there is a bit of competition amongst the friends and not that it’s a “You must do this”, “Who made 8000 today”, “Who made 9000 today” and they give each other badges and pats on the back and so it becomes quite social and that’s quite important I think. We always say if you train with a friend you’re less likely to pull out but if you going to say “I am going to do it on my own” then it is easier.”* |
|  |  | The emotional and social connection maintained between the participants who share the same medical condition. (Lally, 2018) | *“I loved the fact that some of the women [in videos] had the same cancer I did.”* |
|  |  | The interaction with the peers through the program improved the emotional well-being of the patients and reassured feeling of not being alone in the struggle with breast cancer. (Zhu, 2018) | *“I feel better to talk to someone who are in similar situation. Cancer is not a good thing. If I always think about breast cancer alone at home, it is so easy for me to feel bad. I didn’t feel alone when I talked with peers through your program. They might have worse or better conditions than me, but they understand what I meant (Laugh...). This may be the source of comfort and help.”* |
|  |  | The program allowed patients to feel “less alone” in their experience through the interaction between the peers. (Alberts, 2018) | *“Knowing there’s other people taking programs like this and that, it’s kind of, you know, you feel like, well, I’m not alone.”* |
|  |  |  | *“You know that you’re not alone, but when your feelings are validated just by reading someone’s story, I mean that is everything.”* |

Table 7: Theme 4: Meeting individualised patient needs

| **Synthesised themes (third order constructs)** | **Sub-themes** | **Second Order constructs: the authors interpretations of the original findings** | **First Order constructs: examples of direct quotations from the participants of the study** |
| --- | --- | --- | --- |
| **MEETING INDIVIDUALISED PATIENT NEEDS** | ***Timing of the intervention*** | The utility and timing of the intervention to be cancer-specific is uncertain. Amongst the patients, the optimal timing agreed to be dependent on the time of need. (Shaffer *et al.*, 2019) | *“I think it would have been a little easier during radiation. Although I had as much fatigue during radiation… I think it would have been more doable then, for me, possibly.”* |
|  |  |  | *“I had more trouble with sleep issues early on at diagnosis and in between surgeries, so it would have been helpful for me to have enrolled in the program earlier”* |
|  |  |  | *“The very end of your treatment when you finished your chemo and…the doctor says ‘Ok, see you in six months.’ That would be the time to offer it. ‘Cause you feel so unwarned’”* |
|  |  | The appropriate timing of the intervention agreed to be during the first diagnosis in order to reduce feeling overwhelmed and enable information access to the newly diagnosed patients. (Lally *et al.*, 2018) | *“I wish I would have had something like this when I was first diagnosed…I can see this tool being useful in answering questions that have not come to mind when first diagnosed.”* |
|  |  | The patient’s perceived ability to perform a particular task in a low physical or psychological health status reduced the engagement with the mobile application. (Zhu *et al.*, 2018) | “*During the three days hospitalization for chemotherapy, I felt like dying and I couldn’t even think about opening the app. When I came back home and I recovered a little bit, still my health was quite fragile. I couldn’t spend long time reading the app or have enough energy to read in depth.”* |
|  | ***Tailoring to the disease*** | Patients believed the mobile application could be tailored to be better suit the individual’s lifestyle and barriers to achieve the required fitness needs.  (Puszkiewicz *et al.*, 2016) | *“The only thing that holds me back from exercising frequently, is the fatigue, it’s always the fatigue. So […] if an app somehow could consider my fatigue on those bad days. [Because] it really demotivates you… like you know when you just can’t complete a workout because of it.”* |
|  |  |  | *“You can’t put too much pressure on your arms [after lymph node dissection surgery], but you have to train them too to avoid lymphedema. So I think in those terms the application was really good, definitely suitable.”* |
|  |  | The relevance of available information on the program towards the cancer survivors were found unclear. Patients expressed a desire of having a program that is specific to the cancer survivors. (Phillips *et al.*, 2019) | *“I think that it needs to be aimed towards survivors. That would be the first component. There’s a lot on the Internet that gives you a lot of exercises but it’s not aimed towards survivors.”* |
|  |  | The concerns over the treatment side effects limiting the physical activity and the potential adverse effects of the trackers on physical health identified amongst the patients. (Nguyen *et al.*, 2017) | *“Because I’m on medication, and I’ve got the joint issues. So that’s really, I’m really- not struggling but just it stops me doing, you know 5000 I’m all right, if you push me to 6 or 7 [thousand steps], I’m in tears because of the joints”* |
|  |  |  | *“I didn’t like wearing it at night. I didn’t feel comfortable. I wanted to be away from all sort of electrical kinds of things when I sleep. I even have the clock radio quite away and I don’t sleep near any power points or anything. I’ve had breast cancer a few times, it’s always been caught early but you still think you do what you can to keep away from any kind of an influence you think might be affecting you.”* |
|  |  | The advice from the cancer survivors provided trust to the newly diagnosed patients through the comprehensive and accurate content of the program. (Lally *et al.*, 2018) | *“A great tool… because what we need is true information so that we can focus on surviving and I believe that this program would be that tool.”* |
|  | ***Patient suggestions*** | Patients explained a desire of receiving credit from all the daily living activities and a more flexible but tailored program goals to achieve their specific needs. (Phillips *et al.*, 2019) | *“I would want it to track everything and also easily convert all the different kinds of activities into some kind of common measure, so that you could have a total idea of what you did.”* |
|  |  |  | *“I think it has to have different levels. Some people have no idea about how much exercise they should do or what’s useful but then some people…need more information because [they already]…know that. If it’s there and it has different levels available depending on what you need then I would be interested in using it.”* |
|  |  | Suggestions for the content, design and technical improvements of the intervention to overcome barriers to use – for instance, these involved adding a search engine function to instantly locate the information; adding more diet details and specific food recommendations; and addressing the accessibility of the program to help older patients overcome barriers. (Zhu *et al.*, 2018) | *“There are too many content in the Learning forum. I was overwhelmed by the information each time I opened it. I do not have patience to read all of them...But the screen of the mobile phone is so small and it takes long time to find the knowledge you want. The program can be improved by adding search engine in the Learning forum. If I search for “nausea”, then all the knowledge related to nausea will come out. Search engine will help save my time.”* |
|  |  |  | *“Please add more information on the food choice. We need to eat every day, however, there is conflicting advice on food choices on the internet, such as whether we should eat honey, chicken, leek, etc. We are in a dilemma on what we should eat. The apps can provide detailed information on food choice, the time of food intake, the cooking methods, etc... Such practical information would be very helpful.”* |
|  |  |  | *“Some people, like me, 40 or 50 years old. Well, this group believe the apps is a little bit troublesome. They feel challenged to use the new technology. This is a problem. Although they are not willing to participate, they often consulted me on some questions and they were quite interested in the knowledge. If this program can be available for their family members, such as their son or daughter, it would be helpful.”* |
|  |  |  | *“Many women with breast cancer come from the countryside. They are illiterate, or they cannot read and speak Mandarin… if you can open the program to other family members who can read and convey the knowledge to the women, they would also benefit”* |
|  |  | Patients reported the inaccuracy of the devices, as the trackers could not register the light intensity physical activity, and auto-goal function setting adversely affected the patient’s motivation. Adherence to the physical activity found to be influenced by the aesthetics of the trackers. (Nguyen *et al.*, 2017) | *“I looked down, it [the tracker] had a message that said “Move!”. I thought, that’s a bit cheap because I’ve been busy all day working and busy all day, and now I’m finally sitting down and it wants me to move again.”* |
|  |  |  | *“I had the Polar first […] I thought it was quite heavy and quite clunky but then I had the two Garmins and in the end I decided that was my favourite even though it was heavier. I thought it was easier to push the buttons and see where you were rather than the others.”* |
|  |  |  | *“It was a Garmin, say the goal is 10,000 [steps] and you had a lazy morning, it drops down to 8000 and then 6000 and […] “No, I still want to do my 10,000” […] I would rather have the goal set and if I didn’t reach my goal, that’s something I am going to have to deal with but with the goal changing, I could have sat there and the goal would have just dropped, it didn’t seem to be rationale.”* |
|  |  | The opinions varied on cancer-specific tailoring. Some patients would like to have more information on cancer-related insomnia whereas some believed the information would be best as an optional content to avoid distress. (Shaffer *et al.*, 2019) | *“General comments about cancer and insomnia would be really helpful. For me the more information I have the better.”* |
|  |  |  | *“There ought to be a way to bypass, because all cancers are different. Make it possible to bypass if it doesn’t apply to your cancer.”* |
|  |  | The tone of the prompt deemed to be crucial in determining preference and likelihood of use and auto-goal function caused confusion of targeted goal in some patients. (Hardcastle *et al.*, 2018) | *“I’d get a little vibration to say let’s go do 250 steps, it was much more polite than MOVE.”* |
|  |  |  | *“it wasn’t clear like now the goal, does that mean I have to do 5000 steps, why have they got that I’ve been doing over 10000”* |
|  |  | Patients reported the benefits of tele-rehabilitation, complementing it with the occasional in-person visits would improve the knee evaluation by the physiotherapist. (Kairy *et al.*, 2013) | *“I’m fairly certain that at least twice, on two occasions certainly if he would have come, it would have been a plus. Well, maybe psychologically, I think, thinking that he could have manipulated your knee, to see in a tangible manner and be able to manipulate it, but hum… it’s the suggestion that I would give, to at least meet, I don’t know how often.”* |
|  |  | Due to personal barriers and lack of anonymity, some patients were reluctant to actively participate in the online forum. (Das and Faxvaag, 2014) | *“I have reading and writing difficulties as well, so when I start writing, it comes out weird. Then, I become even more reserved regarding writing.”* |
|  |  |  | *“It does not bother me. On other forums, even though you don’t have your name, with a nickname, you can find out who the person is anyway. You have to be very careful if you want to be anonymous.”* |
|  |  | Additional features of including joint angle measure, a quality score after each exercise session and gamification ideas such as unlocking new levels to sustain the engagement were suggested by participants. (Argent *et al.*, 2019) | *“This is probably not possible, but to get the angle of that knee bend, if you knew that… for me that is where I’m really stuck so just to know that… I know it counts it and it said you did it right, but I’m not sure what the angle of the bend is, and I’m rather obsessed with that.”* |
|  |  |  | *“It would be very difficult to rate it from the previous time, there is no linkage from the previous repetitions… So in a way you don’t know if you are doing better today than you did yesterday… The quality of how I’m doing them.”* |
|  |  |  | *“If there was a games element to it you know you have unlocked the next level… or a medal or something.”* |
|  |  | The pinked colour wristbands served as negative reminders of lived breast cancer experiences and created a divide between the survivors and general population. (Kokts-Porietis, 2018) | *“…people are bugged with the [activity trackers being the] colour pink, where other people that’s all they wear is pink. And […] mail correspondents coming in [hospital], or Cancer Society white envelopes is shocking.”* |
|  |  |  | *“Don’t even think about it. It’s like, oh this is just another pink thing”* |
